# Radiation Therapy for Tumor-Induced Ptosis Due to Levator Palpebrae Superioris Dysfunction: A Case Report

**DOI:** 10.7759/cureus.86613

**Published:** 2025-06-23

**Authors:** Yojiro Ishikawa, Rei Kihira, Satoshi Teramura, Kengo Ito, Takayuki Yamada

**Affiliations:** 1 Radiology, Tohoku Medical and Pharmaceutical University, Sendai, JPN; 2 Medicine, Tohoku Medical and Pharmaceutical University, Sendai, JPN

**Keywords:** levator palpebrae superioris, ocular oncology and medical retina, ptosis, radiation therapy, tumor-induced dysfunction

## Abstract

This report presents a unique case of tumor-induced ptosis caused by follicular lymphoma, successfully treated with radiation therapy (RT). A male in his 70s presented with progressive ptosis, swelling of the right upper eyelid, and difficulty opening the right eye. Imaging revealed a well-defined tumor in the right orbit, causing medial displacement of the globe. Magnetic resonance imaging (MRI) showed the tumor to be mildly hyperintense on T2-weighted imaging (T2WI), and positron emission tomography-computed tomography (PET-CT) demonstrated intense fluorodeoxyglucose (FDG) uptake with a maximum standardized uptake value of 17.5. Histopathological analysis confirmed the diagnosis of follicular lymphoma, predominantly Grade 3B (80%) with a component of diffuse large B-cell lymphoma (DLBCL), not otherwise specified (20%). Staging based on the Lugano classification revealed the disease to be Stage IIE due to bilateral cervical lymphadenopathy. The patient declined systemic chemotherapy and opted for localized RT to the right eyelid tumor. The treatment field excluded lymph node metastases, and RT was delivered to a total dose of 36 Gray in 20 fractions. The treatment was well-tolerated, and no significant adverse effects were observed. Tumor regression was evident, and the patient regained the full ability to open the right eye within one month after the treatment. At the one-year follow-up, the tumor in the right upper eyelid had completely disappeared, and the neck region remained stable. This case highlights the efficacy of RT in treating tumor-induced ptosis caused by dysfunction of the levator palpebrae superioris muscle. Radiation therapy remains a valuable therapeutic option, particularly in scenarios where surgery or chemotherapy is not feasible. Further studies are warranted to validate these findings and develop standardized treatment protocols for this distinct condition.

## Introduction

Ptosis is a condition in which the upper eyelid fails to elevate normally due to dysfunction of the levator muscle or its innervating nerves. This condition can significantly impair visual function and negatively affect quality of life (QoL).The prevalence of ptosis in adults is reported to be 0.79 to 1.99 per 10,000 individuals per year [1]; however, this figure may vary depending on the definition and study population. Additionally, post-ophthalmic surgery ptosis has been documented in up to 11.4% of cases, with a particularly high incidence of 5.5% to 5.8% following cataract surgery [2] and 19% after trabeculectomy (glaucoma surgery) [3]. The levator palpebrae superioris muscle plays a crucial role in eyelid elevation and shares a common fascial system with the superior rectus muscle. Anatomically, Whitnall’s ligament (also known as the superior transverse ligament) supports the levator muscle, and the fascial structure between the superior rectus and levator palpebrae superioris is believed to contribute to its stability [4,5].

The etiology of ptosis is diverse, including aging, congenital abnormalities, neuromuscular diseases, trauma, and eyelid tumors. Among these, tumor-induced ptosis is relatively uncommon, and there are limited reports on its management. Eyelid tumors that cause ptosis can originate from various histological subtypes, including benign lesions such as chalazion and neurofibroma, as well as malignant tumors such as sebaceous gland carcinoma, basal cell carcinoma, and lymphoma. Tumors affecting the levator muscle or its nerve supply, such as orbital lymphomas or metastatic lesions, may also contribute to ptosis [6-9]. The standard treatment for tumor-induced ptosis depends on the tumor histology and location, with surgical resection being the primary approach for resectable tumors, while radiotherapy or systemic therapy is considered for unresectable or metastatic cases. This case report describes tumor-related ptosis treated with radiation therapy (RT), resulting in a favorable outcome. It explores the potential effectiveness of RT in the treatment of tumor-induced ptosis.

## Case presentation

**Figure 1:**
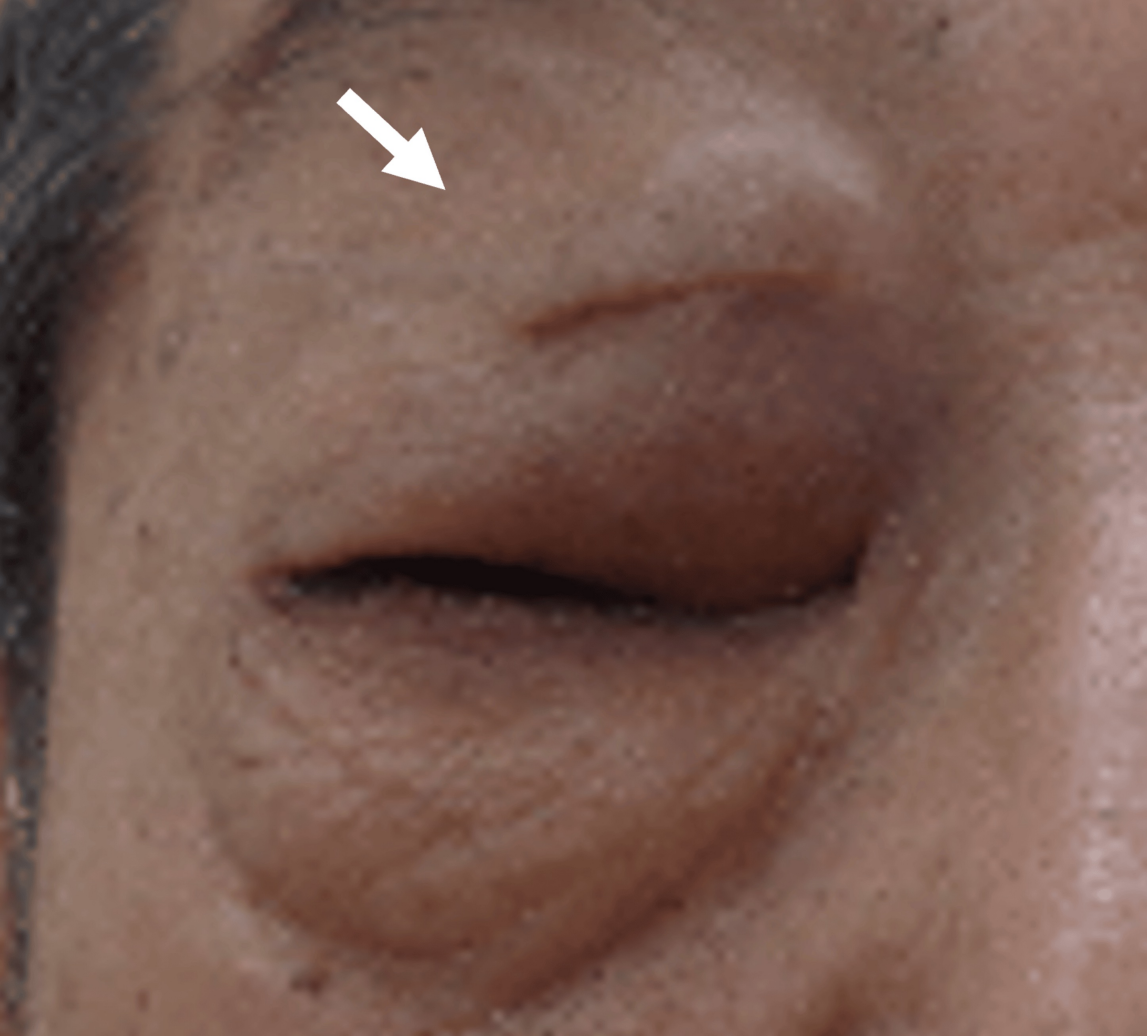
Macroscopic right upper eyelid findings at initial diagnosis Pre-treatment macroscopic examination showed that the patient was unable to open his right upper eyelid due to tumor-induced dysfunction (arrow).

A Japanese male patient in his 70s presented with a one-year history of difficulty opening his right eye initially noted as worsening eyelid drooping upon waking in the morning. The patient reported progressive swelling of the right upper eyelid and impaired ability to fully open the eye, prompting his visit to our hospital's ophthalmology department. He denied any associated pain, visual disturbances, or systemic symptoms such as fever or weight loss. On examination, there was evident swelling and ptosis of the right upper eyelid, with significant difficulty in voluntary eyelid elevation. No conjunctival injection was observed, and both visual acuity and the field of vision remained unaffected. Palpation revealed a firm mass in the superior orbit. The left eye was unaffected, and systemic examination showed no other abnormalities (Figure 1).

**Figure 2:**
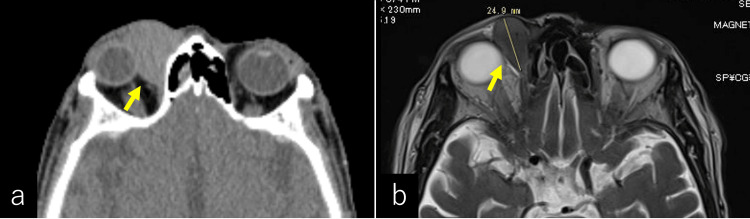
Imaging findings of the right upper eyelid mass at initial diagnosis Computed tomography imaging (a) revealed a well-defined mass, displacing the right eyeball laterally, with bilateral cervical lymphadenopathy. No other definitive tumors or nodules were identified. T2-weighted imaging (T2WI) MRI (b) showed a smooth-edged nodule in the medial right orbit, measuring 24.9 × 21.5 × 30.5 mm in size, with mildly hyperintense signal characteristics.

**Figure 3:**
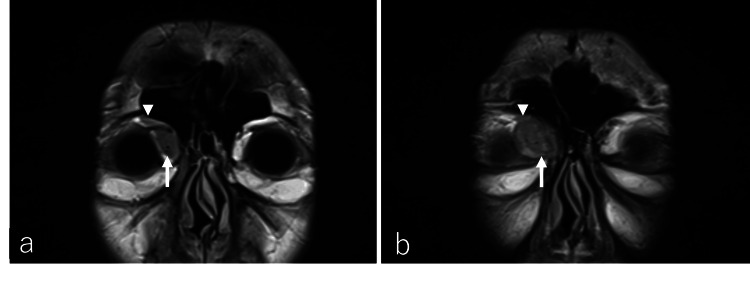
Coronal imaging findings of the right upper eyelid mass at initial diagnosis Coronal fluid-attenuated inversion recovery (FLAIR) images are shown in (a) and (b). The tumor was located in the medial aspect of the orbit (arrow). Its anterior portion extended to the upper eyelid and was in contact with the levator palpebrae superioris muscle (arrowhead).

Computed tomography (CT) of the orbit revealed a well-circumscribed, smooth-edged mass located in the medial aspect of the right orbit (Figure 2a). The lesion displaced the globe laterally without evidence of bone erosion. Subsequent magnetic resonance imaging (MRI) provided further characterization of the lesion. On T2-weighted imaging (T2WI), the mass exhibited a mildly hyperintense signal and measured 24.9 × 21.5 × 30.5 mm in size (Figure 2b), while it appeared isointense on T1-weighted imaging (T1WI). The tumor extended to the eyelid and was in contact with the levator palpebrae superioris muscle (Figures 3a-3b). Diffusion-weighted imaging (DWI) showed a hyperintense signal with a corresponding reduction in the apparent diffusion coefficient (ADC), suggesting restricted diffusion. Additionally, multiple enlarged lymph nodes were detected bilaterally in the cervical region.

**Figure 4:**
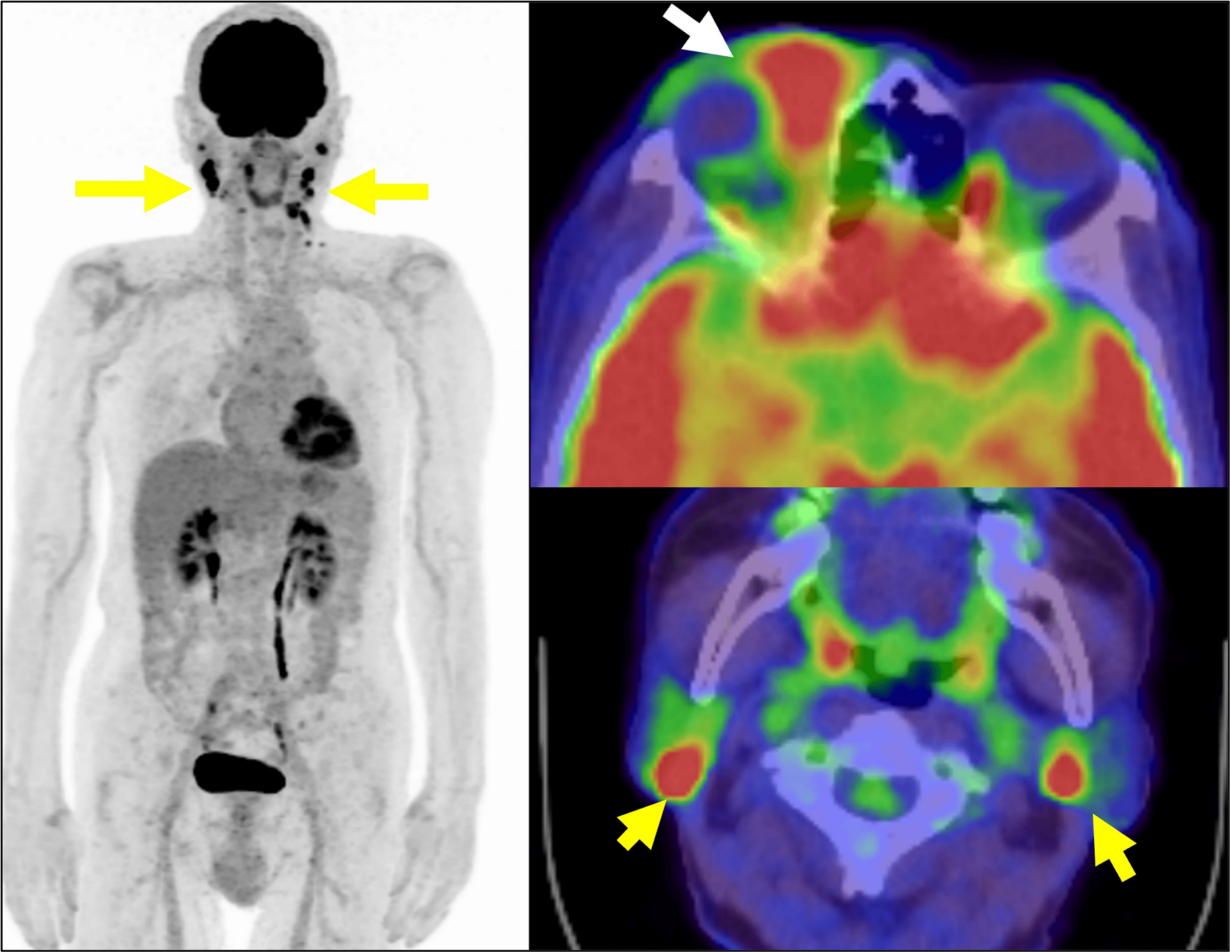
PET-CT findings of the right upper eyelid mass and bilateral cervical lymph nodes at initial diagnosis Positron emission tomography-computed tomography (PET-CT) imaging revealed intense fluorodeoxyglucose  (FDG) uptake in the right orbital mass (white arrow) and bilateral cervical lymph nodes (yellow arrows), with a maximum standardized uptake value (SUVmax) of 17.5. No other significant lymph node enlargement or abnormal FDG uptake was identified in other regions.

Positron emission tomography-computed tomography (PET-CT) demonstrated a high standardized uptake value (SUVmax) uptake (17.5) in the right orbital mass and cervical lymph nodes, consistent with metabolic activity suggestive of malignancy (Figure 4).

**Figure 5:**
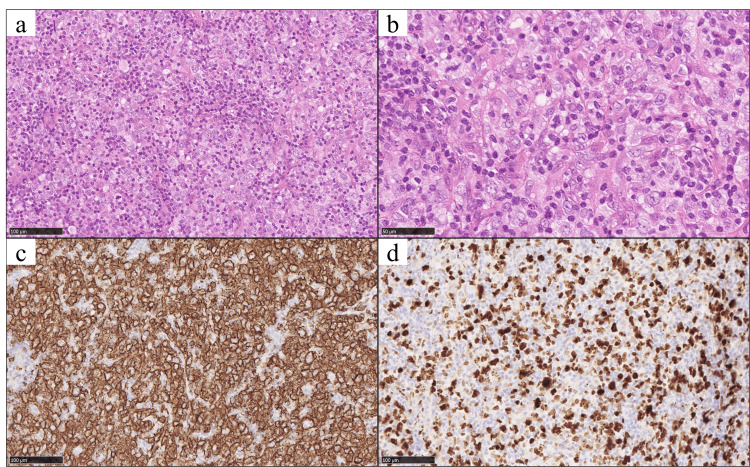
Histopathological and immunohistochemical findings of the orbital tumor (a) Hematoxylin and eosin (H&E) staining, low magnification (20x): The tumor displayed densely packed atypical cells with nuclear irregularity and abundant eosinophilic cytoplasm. (b) H&E staining, high magnification (40x): Prominent mitotic figures were observed, with interspersed small lymphocytes. (c) Immunohistochemical staining for CD20 (20x): Strong membranous positivity confirmed B-cell lineage. (d) Ki-67 staining (20x): Approximately 60% of tumor cells showed positive nuclear staining, indicating a high proliferation index.

Histopathological examination of the orbital tumor revealed densely packed large atypical cells with prominent nuclear irregularity and abundant eosinophilic cytoplasm. Frequent mitotic figures were observed, with small lymphocytes interspersed around the atypical cells (Figures 5a-5b). Immunohistochemical analysis demonstrated positive staining for CD20, CD45, and CD79a, confirming the tumor's B-cell lineage. Ki-67 labeling indicated a proliferation index of approximately 60% (Figures 5c-5d), supporting a highly proliferative nature. Weak positivity for NUM1, Bcl-2, and Bcl-6 was noted, while CD3, CD5, CD10, CD56, CD68, and AE1/AE3 were negative, ruling out T-cell or epithelial origins. Follicular dendritic cell markers CD21, CD23, and CD35 showed variable expression, consistent with disorganized follicular structures. The tumor was composed of approximately 80% follicular lymphoma (Grade 3B) and 20% diffuse large B-cell lymphoma (DLBCL), not otherwise specified. 

**Figure 6:**
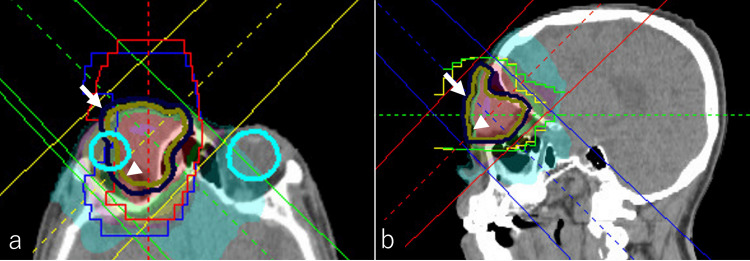
Radiation therapy planning for the right upper eyelid mass The radiation therapy plan for the treatment of the right orbital mass is illustrated in axial (a) and sagittal (b) views. The gross tumor volume (GTV) was defined as the localized tumor in the right orbit. The clinical target volume (CTV) was created by adding a 5-mm margin to the GTV (arrowhead) with anatomical adjustments. The planning target volume (PTV) was further expanded by adding a 3-mm margin to the CTV for treatment planning purposes (arrow). The treatment plan was developed using the RayStation system (RaySearch Laboratories, Stockholm, Sweden), employing a non-opposed four-field technique to optimize dose distribution. A total dose of 36 Gy was delivered in 20 fractions using 4MV X-rays.

Staging based on the Lugano classification revealed the disease to be Stage IIE due to bilateral cervical lymphadenopathy. As the lymphoma was classified as Grade 3B, systemic chemotherapy was considered. However, the patient declined systemic chemotherapy and hospitalization, citing personal preferences. Therefore, localized RT was pursued to achieve symptom relief and local disease control. If the disease had been more localized, lower radiation doses such as 24 Gy or 4 Gy could have been considered. However, given the presence of Grade 3B lymphoma and DLBCL components, as well as the patient’s refusal of systemic chemotherapy, a total dose of 36 Gy was selected. A total dose of 36 Gy was administered in 20 fractions using a 4MV X-ray beam. The gross tumor volume (GTV) encompassed the right orbital mass, with a 5-mm margin added to define the clinical target volume (CTV). Planning target volume (PTV) was created by adding a 3-mm margin to the CTV. Treatment planning was performed using RayStation software (RaySearch Laboratories, Stockholm, Sweden), employing a four-field technique to optimize dose distribution and minimize exposure to adjacent organs at risk (OARs), including the contralateral orbit and optic nerve (Figures 6a-6b). No treatment was administered to the cervical lymph nodes.

**Figure 7:**
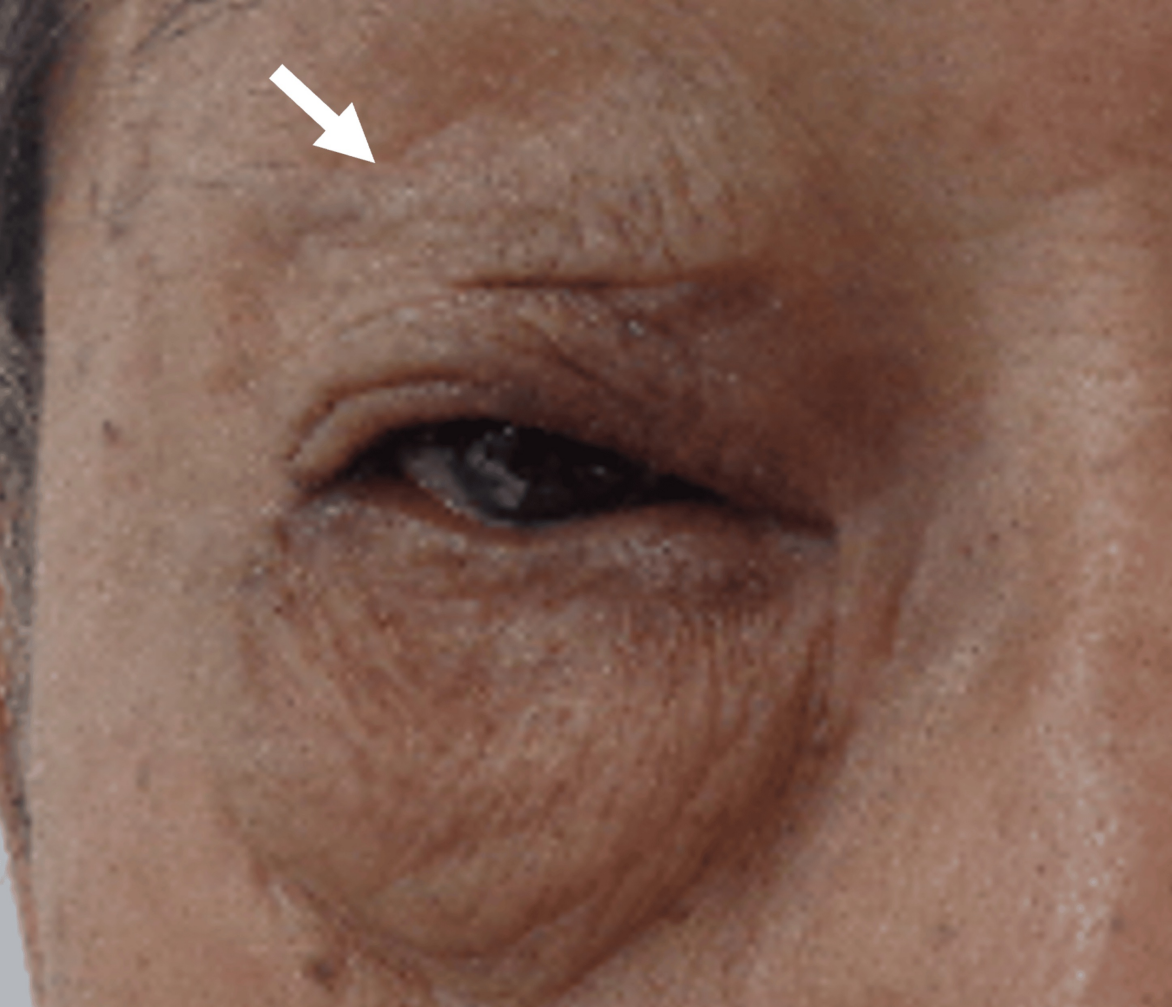
Macroscopic findings of the right upper eyelid after radiation therapy Post-treatment macroscopic examination shows that the patient regained the ability to open his right eye (arrow). No significant adverse events were observed during radiation therapy. Tumor regression was noted during the course of treatment, and complete resolution of ptosis was achieved one month after completing radiation therapy.

No significant acute toxicities were observed during the course of RT. Clinical improvement was noted as early as two weeks after treatment initiation. At the one-month follow-up, the patient reported the ability to fully open his right eye, with a significant reduction in swelling and ptosis (Figure 7). Computed tomography imaging at this time confirmed marked tumor shrinkage. At the one-year follow-up, the patient remained in complete remission (CR) of the orbital lesion with no recurrence.

## Discussion

The relationship between tumors and ptosis has been investigated in several studies, revealing a strong association between tumors affecting the nervous system or central nervous system and the pathology of ptosis. Tumors such as malignant peripheral nerve sheath tumors (MPNST), pituitary tumors, and hematologic malignancies like acute lymphoblastic leukemia and neuroblastoma have been reported as causes of ptosis [6-9]. For instance, in a case involving a four-year-old girl with left ptosis, a mediastinal tumor was identified and diagnosed as MPNST, causing nerve compression and resulting in functional impairment [6]. Similarly, Yen et al. emphasized that ptosis can serve as an early manifestation of pituitary tumors, highlighting the importance of recognizing this symptom for timely diagnosis [7]. Hematologic malignancies have also been implicated in ptosis, often related to neurotoxicity from chemotherapy or direct involvement of the central nervous system [8,9]. Treatment strategies for tumor-induced ptosis vary depending on the underlying tumor type and pathology. For benign or resectable tumors, surgical excision may be considered if the lesion directly compresses the levator palpebrae superioris muscle or its innervating structures. Specific ptosis treatment options include Müller muscle-conjunctival resection, levator shortening, or frontalis sling procedures, tailored to the severity and underlying etiology [10].

Radiation therapy has emerged as a highly effective treatment option for eyelid lymphomas, which are rare malignancies primarily comprising non-Hodgkin lymphoma (NHL) and mucosa-associated lymphoid tissue (MALT) lymphoma. Moderate-dose radiation therapy, typically in the range of 24 to 30 Gy, achieves local control rates exceeding 95% for MALT lymphomas, while low-dose therapy at 24 Gy demonstrates a 100% complete response rate for orbital lymphomas, maintaining high local control rates over extended follow-up periods. Additionally, follicular lymphoma can be effectively treated with lower doses, such as 4 Gy, based on recent clinical evidence [11,12]. Prognosis varies depending on histological subtypes. Generally, extranodal marginal zone lymphoma (EMZL), follicular lymphoma (FL), and early-stage mycosis fungoides (MF) are associated with favorable outcomes, whereas diffuse large B-cell lymphoma (DLBCL) and mantle cell lymphoma (MCL) tend to have poorer prognoses [13]. Furthermore, radiation therapy has demonstrated effectiveness in addressing tumor-related complications in other cases, including conjunctival and orbital lymphoid tumors, by reducing compression-induced symptoms [14,15].

In the present case, ptosis was caused by a lymphoma that predominantly consisted of follicular lymphoma elements with a minor component of DLBCL. The tumor exerted significant compression on the levator palpebrae superioris muscle, leading to functional impairment. This aligns with the broader concept of tumor-associated muscle dysfunction. Specifically, the mechanical and neurological effects observed in this case are consistent with the mechanisms described in other reports of tumor-induced muscle dysfunction [16-18]. Radiation therapy effectively reduced the tumor volume, alleviating the compression and restoring muscle function. Given the mixed histology of the lymphoma, including DLBCL and Grade 3B follicular lymphoma, RT was tailored to deliver 36 Gy in 20 fractions, slightly exceeding the standard dose range for follicular or MALT lymphomas. This tailored approach provided effective disease control while highlighting the versatility of radiation therapy in managing tumors with mixed histological components. However, if the tumor had consisted solely of follicular lymphoma and had been more localized, a lower total dose, such as 24 Gy or even 4 Gy, may have been a more appropriate option [11,13]. The increased dose emphasizes the importance of careful long-term monitoring for potential complications such as cataracts and chronic dry eye, particularly in anatomically sensitive areas like the eyelid [14,15]. 

To further explore the concept of tumor-associated muscle dysfunction [16-18], it may be beneficial to consider tumor-induced ptosis, particularly cases involving compression of the levator palpebrae superioris muscle, within this broader framework. Recognizing these conditions collectively could provide valuable insights into their pathophysiology and management. This case, with its mixed histology and favorable response to RT, suggests that tumor-induced ptosis might fit within this category. Further studies, including systematic reviews and clinical trials, would be valuable in refining the classification, diagnosis, and treatment approaches for such conditions. Advanced techniques such as intensity-modulated RT, proton beam therapy, and tomotherapy, along with protective measures like lens shielding, could further optimize treatment outcomes by minimizing risks while maintaining therapeutic efficacy [14,15,19,20].

## Conclusions

This case highlights the efficacy of RT in treating tumor-induced ptosis caused by dysfunction of the levator palpebrae superioris muscle, demonstrating its ability to reduce tumor volume and alleviate symptoms. Radiation therapy remains a valuable therapeutic option, particularly in cases where surgery or chemotherapy is not feasible. Although tumor-induced ptosis is rare, considering tumor-induced levator palpebrae superioris dysfunction in the diagnostic and treatment process is crucial for accurate clinical assessment and appropriate management. Recognizing this condition as a distinct clinical entity may contribute to improved diagnostic accuracy and guide tailored treatment strategies. Further studies and additional case reports are warranted to validate these findings and establish standardized treatment protocols.
